# 

**Published:** 1899-12-09

**Authors:** 


					ma nvari rxu. " 'me standard ot nignesr puniy,"?Lancet.
"December"vitti isyy.
CAD??RYS t H ? CADc^PY's
ABSOLUTELY PURE. ^ NO DRUGS OR ALKALIES USED.
ygjfgt. - ^
fCLAScow Western Infirmar
No. m Vol. XXVII. [SSdpear!] Saturday,
Dec. 9th, 1899.
pubscriptonTcnns,] pK(ICE TWOPENCE.

				

